# Zafirlukast induces DNA condensation and has bactericidal effect on replicating *Mycobacterium abscessus*

**DOI:** 10.1128/aac.00029-24

**Published:** 2024-07-11

**Authors:** Sanne van der Niet, Keith D. Green, Irene M. Schimmel, Jordy de Bakker, Bastiaan Lodder, Eric A. Reits, Sylvie Garneau-Tsodikova, Nicole N. van der Wel

**Affiliations:** 1 Electron Microscopy Centre Amsterdam, Amsterdam University Medical Centre, Amsterdam, the Netherlands; 2 College of Pharmacy, University of Kentucky, Lexington, Kentucky, USA; St. George's University of London, London, United Kingdom

**Keywords:** *Mycobacterium abscessus*, antibiotics, zafirlukast, electron microscopy

## Abstract

*Mycobacterium abscessus* infections are emerging in cystic fibrosis patients, and treatment success rate in these patients is only 33% due to extreme antibiotic resistance. Thus, new treatment options are essential. An interesting target could be Lsr2, a nucleoid-associated protein involved in mycobacterial virulence. Zafirlukast is a Food and Drug Administration (FDA)-approved drug against asthma that was shown to bind Lsr2. In this study, zafirlukast treatment is shown to reduce *M. abscessus* growth, with a minimal inhibitory concentration of 16 µM and a bactericidal concentration of 64 µM in replicating bacteria only. As an initial response, DNA condensation, a known stress response of mycobacteria, occurs after 1 h of treatment with zafirlukast. During continued zafirlukast treatment, the morphology of the bacteria alters and the structural integrity of the bacteria is lost. After 4 days of treatment, reduced viability is measured in different culture media, and growth of *M. abscessus* is reduced in a dose-dependent manner. Using transmission electron microscopy, we demonstrated that the hydrophobic multilayered cell wall and periplasm are disorganized and ribosomes are reduced in size and relocalized. In summary, our data demonstrate that zafirlukast alters the morphology of *M. abscessus* and is bactericidal at 64 µM. The bactericidal concentration of zafirlukast is relatively high, and it is only effective on replicating bacteria but as zafirlukast is an FDA-approved drug, and currently used as an anti-asthma treatment, it could be an interesting drug to further study in *in vivo* experiments to determine whether it could be used as an antibiotic for *M. abscessus* infections.

## INTRODUCTION

*Mycobacterium abscessus* is a rapidly growing member of the non-tuberculous mycobacteria (NTM), which causes infections in soft tissues and pulmonary infections. The prevalence of *M. abscessus* pulmonary infections in cystic fibrosis patients is increasing, while treatment success rate of pulmonary disease is only 33%, resulting in increased mortality among these patients (1, 2). As *M. abscessus* can survive in human water sources (i.e., hospital and domestic water supplies) (3) and can be transmitted through human contact (4), numerous potential infection sources are present. However, the exact transmission routes remain elusive.

*M. abscessus* is considered to be one of the most antibiotic-resistant of all mycobacteria, as these mycobacteria have developed multiple intrinsic resistance mechanisms (5, 6), such as a hydrophobic multilayered cell wall, making it difficult for antibiotics to enter, as well as antibiotic efflux pumps and enzymes that neutralize antibiotics in the cytoplasm (5, 6). These efflux pumps are upregulated upon antibiotic stress (7). Macrolide-based antibiotics are generally used for NTM; however, *M. abscessus* has inducible resistance through expression of the *erm(41*) gene which can block binding of macrolides to the ribosome (8). Therefore, treatment with at least three active drugs is advised in the initial phase of treatment, depending on susceptibility assays (9). Due to these resistance mechanisms, the optimal therapy and duration of treatment are not standardized, and treatment needs to be constantly optimized. Taken together, there is a great need for new antibiotic targets to treat *M. abscessus*.

A potential new drug target for *M. abscessus* includes nucleoid-associated proteins (NAPs). Bacteria can condense their DNA in response to environmental stress (10). In *Escherichia coli*, stress-induced DNA-condensation was shown to induce homology-driven repair of DNA double-strand breaks, which demonstrated a role for nucleoid condensation in protecting the genome during stress (11). In addition to stress conditions, *Mycobacterium smegmatis* condenses its DNA upon quiescence (12, 13). This leads to reduced metabolism and increased tolerance to stress and antibiotics. We have previously shown that DNA condensation upon antibiotic stress is also observed in mycobacteria (14) and that inhibiting DNA acetylation is effective in inhibiting recovery from antibiotic-induced DNA condensation, resulting in increased mycobacterial killing (14).

If DNA condensation is a potent drug target, can blocking the function of DNA-binding proteins lead to increased bacterial mortality? A potential target could be Lsr2, which is a highly conserved NAP in mycobacterial species and has a histone-like function (15, 16). It binds AT-rich DNA regions and is able to form DNA bridges (17–19). It has been shown that Lsr2 binds around 21% of the *Mycobacterium tuberculosis* genes (19) and protects mycobacterial DNA from oxidative damage and antibiotic stress, making Lsr2 crucial for *M. tuberculosis* viability (16, 20–22). In *M. smegmatis*, a lack of Lsr2 causes abnormal bacterial colony morphology and affects DNA replication duration and dynamics (23, 24). In *M. abscessus*, which forms both smooth and rough colonies (25, 26), the rough variant is more virulent, has higher Lsr2 transcription levels, has a lower glycopeptidolipid content in the cell wall, and has increased antibiotic resistance compared to the smooth variant (26–29). In addition, a significant reduction in survival of ∆Lsr2 *M. abscessus* is observed in both murine macrophages, zebrafish, and lungs of Balb/c mice compared to wild type (27). These studies indicate that Lsr2 plays an important role in mycobacterial cell cycling and survival both *in vitro* and *in vivo*. In a large drug screening effort, Pinault et al. (30) discovered that the anti-asthma drug zafirlukast (ZAF) targets Lsr2. This cysteinyl leukotriene receptor antagonist is able to block the interaction between Lsr2 and mycobacterial DNA, which occurs through direct and dose-dependent binding (30). As ZAF is an FDA-approved compound safely used to treat asthma, it could potentially be repurposed as a potential treatment for *M. abscessus* infection, given that these mycobacteria are so difficult to treat.

In the current study, we analyzed the effects of ZAF on survival and morphology of *M. abscessus*. We determined the effects of different concentrations ZAF on *M. abscessus* survival over time. In addition, DNA organization and condensation were monitored by imaging of ZAF-treated and ZAF-untreated *M. abscessus* over time. Furthermore, changes in the morphology of *M. abscessus* DNA and cell wall were studied using high-resolution transmission electron microscopy (TEM).

## RESULTS

### ZAF reduces the growth of *M. abscessus*

Lsr2 is highly conserved among mycobacterial species; it is essential for the viability of *M. tuberculosis* and plays an important role in the survival of *M. abscessus* in zebrafish and mice (16, 20–22, 24, 27). Because ZAF might affect mycobacteria other than *M. tuberculosis* (30), the effect of this compound was tested in a minimal inhibitory concentration (MIC) screen on *M. abscessus*, *Mycobacterium avium* and *Mycobacterium intracellulare*. The ability of ZAF to bind albumin with high specificity has previously been demonstrated (31), and thus, ZAF function was tested both in culture medium with and without albumin. Mycobacteria were grown for 3 days to 3 weeks, depending on the mycobacterial growth curve. For *M. abscessus* cultured in MH2 medium, an MIC value of 16 µM ZAF was observed (Table 1). However, when *M. abscessus* was cultured in 7H9 medium or 7H9 medium supplemented with albumin, dextrose, and catalase (ADC), the MIC was greater than 128 µM ZAF, indicating that the composition of the culture medium affects efficacy of ZAF. We also tested five other previously published ZAF derivatives that were shown to have improved activity over ZAF in *Porphyromonas gingivalis* (32). Unfortunately, in this system, these compounds did not inhibit the growth of any mycobacteria (Table 1). Kanamycin A (KAN) and amikacin (AMK) were used as a known anti-mycobacterial controls. The MIC values of ZAF against *M. avium* and *M. intracellulare* were greater than 128 µM, demonstrating that ZAF was not efficient in inhibiting the growth of these mycobacteria. To determine if ZAF affects other non-mycobacterial species, cultures of *Salmonella enterica*, *Klebsiella pneumoniae*, *Acinetobacter baumannii*, *E. coli*, *Pseudomonas aeruginosa*, and *Enterobacter cloacae* were tested, but all had a MIC value higher than 128 µM, whereas the cultures did respond to KAN and AMK (Table S1). In summary, ZAF can reduce the growth of *M. abscessus*, and as *M. abscessu*s has urgent clinical applications, we decided to focus on the effect of ZAF on *M. abscessus*.

**TABLE 1:** MIC values (µM) of ZAF and its synthesized analogs tested against different mycobacterial species

	*M. abscessus* ATCC 19977			*M. avium* ATCC 25291		*M. intracelluare* ATCC 13950
Medium	MH2	7H9	7H9 + ADC	7H9	7H9 + ADC	7H9 + ADC
Compound						
ZAF	16	>128	>128	>128	>128	>128
SGT833	>128	>128	>128	>128	>128	>128
SGT711	>128	>128	>128	>128	>128	>128
SGT710	>128	>128	>128	>128	>128	>128
SGT715	>128	>128	>128	>128	>128	>128
SGT716	>128	>128	>128	>128	>128	>128
KAN	<0.5	<0.5	<0.5	<0.5	<0.5	<0.5
AMK	0.1	<0.5	<0.5	<0.5	<0.5	<0.5

### ZAF reduces the growth of *M. abscessus* in a dose-dependent manner

**Fig 1:**
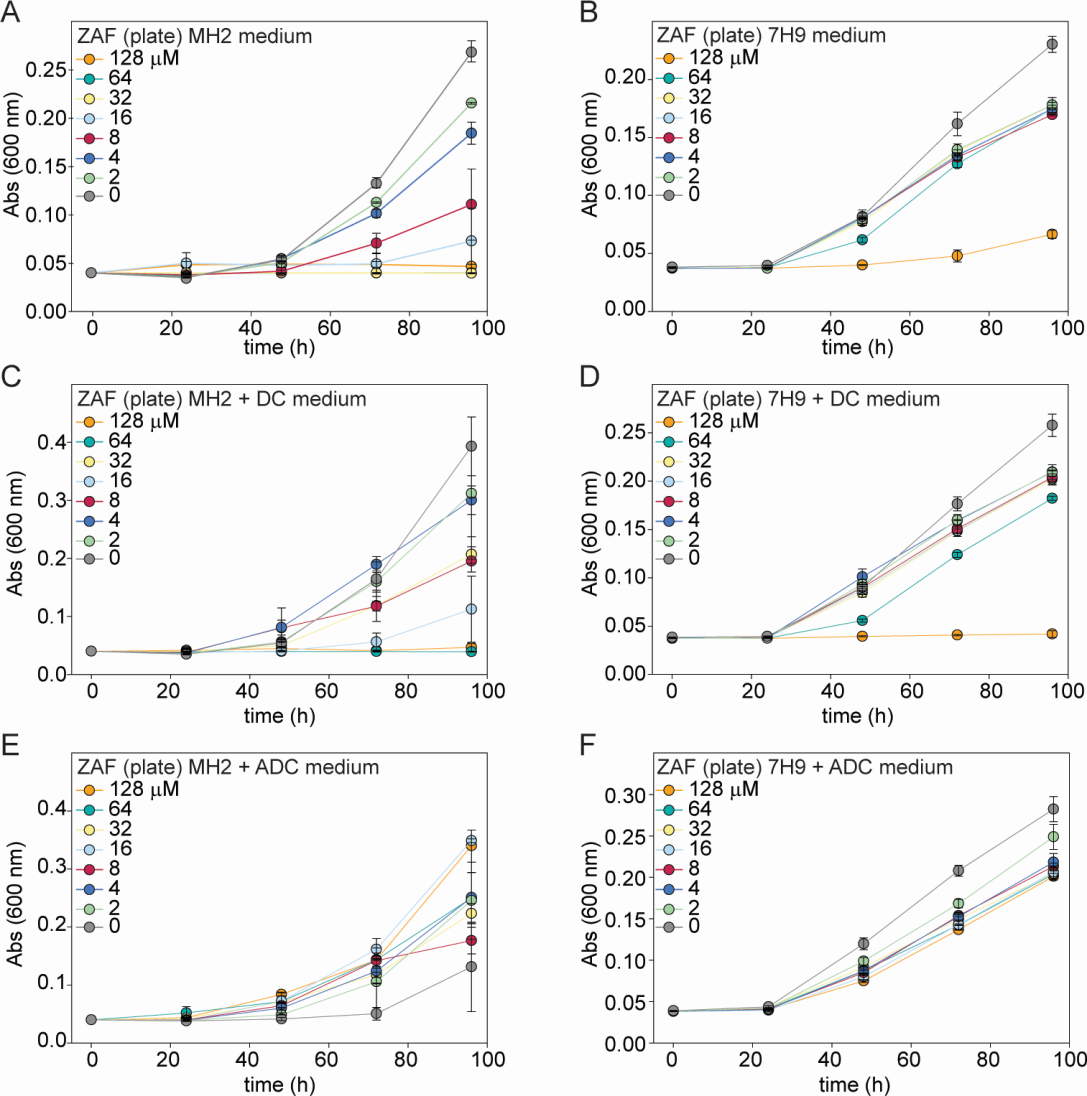
Growth curves of ZAF-treated *M. abscessus* in different culture media. *M. abscessus* was treated with 2 to 128 µM ZAF, and absorption was measured every 24 h for a period of 96 h. Bacteria were cultured in (**A**) MH2 medium, (**B**) 7H9 medium, (**C**) MH2 medium supplemented with DC, (**D**) 7H9 medium supplemented with DC, (**E**) MH2 medium supplemented with ADC, and (**F**) 7H9 medium supplemented with DC. Graphs represent the mean of two individual experiments, and the error bars represent the standard error of the mean. Abs(600 nm) is 600-nm absorbance value.

To gain more insight into the effect of ZAF on *M. abscessus*, we studied the effects of incubation time and dosage on growth. Bacterial cultures treated with ZAF concentrations ranging from 2 to 128 µM for 96 h, and the optical density was measured every 24 h (Fig. 1). In Fig. S1 and S2, time points 72 h and 96 h are shown separately, respectively. When cultured in MH2 medium, a microbiological growth medium regularly used for antibiotic susceptibility testing, a reduction in *M. abscessus* growth was observed after 48 h in a dose-dependent manner (Fig. 1A). The effect of ZAF on growth rates became more pronounced at 72 and 96 h when treated with 2 to 16 µM ZAF in a dose-dependent manner (Fig. S1A, S2A, S3A and B; Fig. 1A). At 96 h, a significant reduction in OD_600_ was observed for all ZAF concentrations above 2 µM, except for 8 µM where a variable response between the replicate experiments was detected (Fig. 1A). For *M. abscessus* cultured in MH2 medium, ZAF concentrations of 32, 64, and 128 µM, appeared to be bacteriostatic (Fig. 1A). The effect of ZAF was less pronounced when *M. abscessus* was cultured in 7H9 medium (Fig. 1B). At 96 h, a non-significant reduction of 25% in growth was observed when treated with 2 to 64 µM ZAF, with no dose-dependent effect observed (Fig. 1B). A significant reduction in *M. abscessus* growth was only established with the maximum concentration of 128 µM ZAF (Fig. 1B).

As mycobacteria are often grown in MH2 medium supplemented with dextrose and catalase (DC), these culture conditions were also tested. As expected, these cultures grew to a higher OD_600_ (0.4 versus 0.25 in unsupplemented medium) (Fig. 1A and C). In these cultures, a non-significant reduction in *M. abscessus* growth was observed when treated with 2 to 32 µM ZAF, and a significant reduction in growth was achieved with 64- and 128 µM ZAF in MH2 + DC medium (Fig. 1C). These data demonstrate that a higher dose of ZAF is needed to reduce *M. abscessus* growth when DC is added to MH2 medium. In 7H9 medium, the addition of DC resulted in a small yet significant reduction of *M. abscessus* growth at 96 h with 2 to 128 µM ZAF (with the exception of 4 µM) (Fig. 1D). As expected, the addition of albumin in combination with DC reduced the effect of high concentrations of ZAF on *M. abscessus* growth in 7H9 medium; however, the reduction in growth rates is still significant (Fig. 1F). In MH2 medium, the effect of ZAF on *M. abscessus* growth was completely abolished by the addition of ADC (Fig. 1E).

To determine if ZAF has a synergistic effect on bacterial growth rates in combination with other antibiotics, we tested the MIC of ZAF in combination with cefotaxamine, imipenem (IPM), kanamycin, rifampicin (RIF), and tigecyline (Fig. S4). Under MH2 culture conditions, there was no reduction in the MIC values of ZAF when combined with these antibiotics. In summary, in MH2 medium without albumin supplement, ZAF reduced the growth of *M. abscessus* after 48 h in a dose-dependent manner. However, culture conditions affected the efficacy of ZAF, and no synergistic effect with other antibiotics was observed.

### Bactericidal effect of ZAF on replicating *M. abscessus*

**Fig 2:**
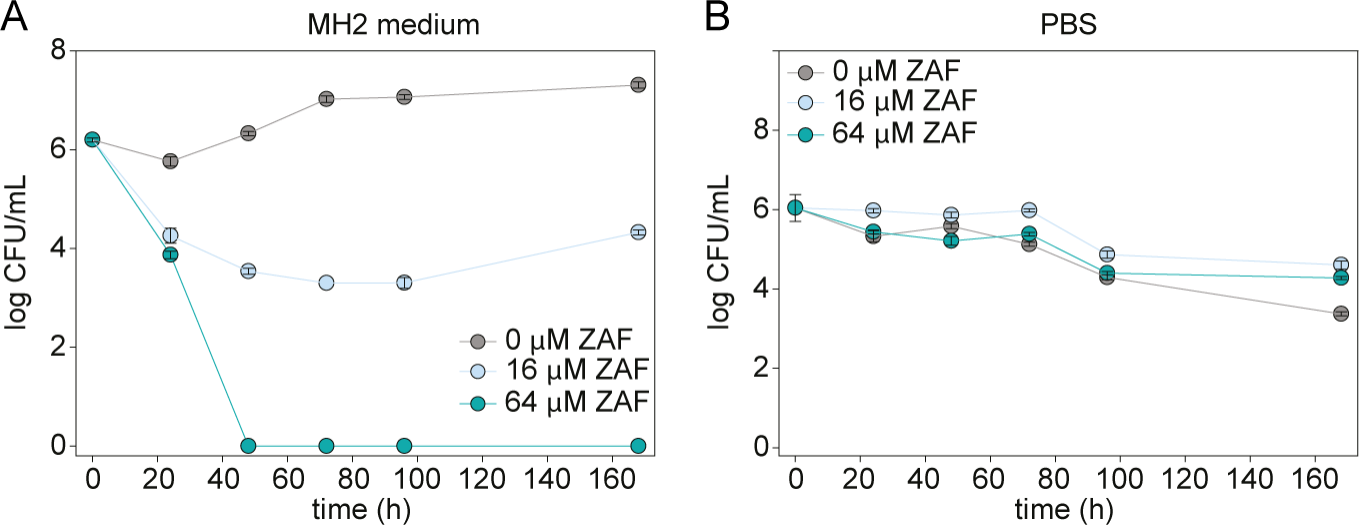
CFU of *M. absessus* treated with ZAF. (**A**) *M. abscessus* was grown in MH2 medium and treated with 0 (gray)-, 16 (light blue)-, and 64 (turquoise) µM ZAF. CFU were determined at 24, 48, 72, 96, and 168 h. (**B**) *M. abscessus* was grown in PBS to achieve a non-replicating state and was treated with 0 (gray)-, 16 (light blue)-, and 64 (turquoise) µM ZAF. CFU were determined at 24, 48, 72, 96, and 168 h. Error bars represent the variation of two independent cultures.

To understand if ZAF simply slows the growth of *M. abscessus* or kills the bacteria completely, the number of viable cells, determined as colony-forming units (CFU), was measured for *M. abscessus* treated with 0, 16 (1 × MIC), and 64 µM ZAF (4 × MIC). As seen in Fig. 2A, treatment of *M. abscessus* with 16 µM ZAF reduced the number of viable bacteria by approximately 2 orders of magnitude compared to untreated cultures. Interestingly, *M. abscessus* treated with 64 µM ZAF was completely killed after 2 days of incubation. We also wanted to know if the ZAF treatment would work on non-replicating *M. abscessus*. For this experiment, a previously published study was followed where it was shown that incubating *M. abscessus* in phosphate-buffered saline (PBS) would result in non-replicating bacteria (i.e., the CFU/mL stays relatively the same over the incubation period) (33). As seen in Fig. 2B, the treatment of non-replicating *M. abscessus* with ZAF does not significantly affect the CFU/mL observed.

### Treatment with ZAF causes early DNA condensation

**Fig 3:**
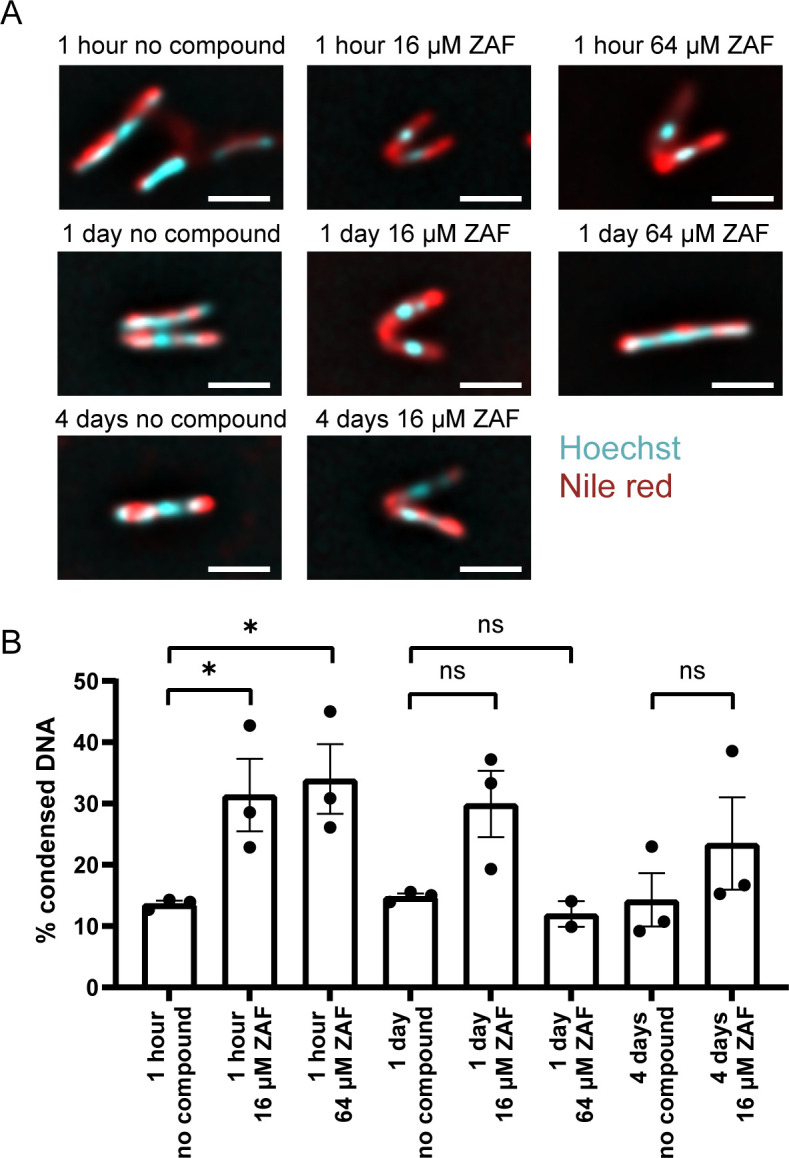
ZAF induces rapid DNA condensation. (**A**) Representative deconvoluted widefield fluorescent images of *M. abscessus* treated with no compound, 16 or 64 µM ZAF for 1 h, 1 day, and 4 days. DNA is stained with Hoechst (cyan) and lipids with Nile red (red). Scale bars represent 2 µm. (**B**) Quantification of DNA pattern of *M. abscessus* in *n* = 3 experiments and *n* = 2 experiments for 64 µM day 1. DNA was defined as condensed when it was present in one compact nucleoid. Per condition, 40–479 *M*. *abscessus* were analyzed. *P < 0.05, and error bars represent the standard error of the mean.

As previous research showed that ZAF prevents the binding of Lsr2 to mycobacterial DNA (30) and Lsr2 is a DNA binding protein (17–19), we studied the effect of ZAF on DNA organization in *M. abscessus*. In order to visualize the DNA, fluorescent staining of the DNA using Hoechst was performed. To determine the outline of the bacterium, Nile red marking lipids were used (Fig. 3A; Fig. S5) similar to the method we previously published (14). The percentage of *M. abscessus* with condensed DNA was determined (Fig. 3B). DNA was considered condensed when it was organized as one small nucleoid. To gain insight into the dynamics of DNA organization, multiple time points were imaged, starting with 1 h of ZAF treatment, to determine a primary response of *M. abscessus* on ZAF, till day 4, as the OD_600_ curves clearly showed the effect of ZAF (Fig. 1A). The concentrations of 16 and 64 µM ZAF were used as a medium and high dose of ZAF. At day 4, *M. abscessus* cultures treated with 64 µM ZAF were very dilute, and no bacteria were visible. This is in agreement with the CFU data (Fig. 2A), showing no surviving bacteria after 48 h of 64 µM ZAF treatment.

At 1 h, day 1, and day 4 of untreated *M. abscessus*, DNA was mainly dispersed throughout the whole cell, indicating that the culture conditions did not affect DNA organization over time. At 1 h, both 16 and 64 µM ZAF caused a significant increase in DNA condensation (Fig. 3B). When DNA was condensed, it was visible as a small dense nucleoid (Fig. 3A). The average percentage of bacteria with condensed DNA was 31% when treated with 16 µM ZAF and 34% when treated with 64 µM ZAF, compared to 14% in untreated *M. abscessus*. We previously showed that *M. tuberculosis* and *M. smegmatis* condense their DNA within 10 min of exposure to antibiotics (14). The fact that ZAF induces DNA condensation in *M. abscessus* indicates that these mycobacteria are stressed by the treatment. After 1 day of ZAF treatment, the percentage of DNA condensation was still elevated when treated with 16 µM ZAF in all three experiments, with an average of 30% compared to 15% in untreated *M. abscessus*; however, this increase was not significant (Fig. 3B). Also, treatment with 64 µM ZAF for 1 day did not result in elevated DNA condensation with an average of 12% in both experiments (Fig. 3A and B). We have shown previously that DNA condensation is a rapid stress response in live and not dead *M. smegmatis* (14). In *M. abscessus* cultures, at days 1 and 4 of 64 µM ZAF treatment, DNA condensation was no longer evident (Fig. 3B), possibly due to the fact that these bacteria did not survive the ZAF treatment; this is consistent with the reduced number of viable cells as seen with the CFU data (Fig. 2A). Thus, DNA condensation occurs rapidly under ZAF treatment in *M. abscessus* and, at later stages, the localization of the DNA changes similar to the dispersed DNA described before in dying *M. smegmatis* (14).

### High-resolution imaging of ZAF induced DNA condensation

**Fig 4:**
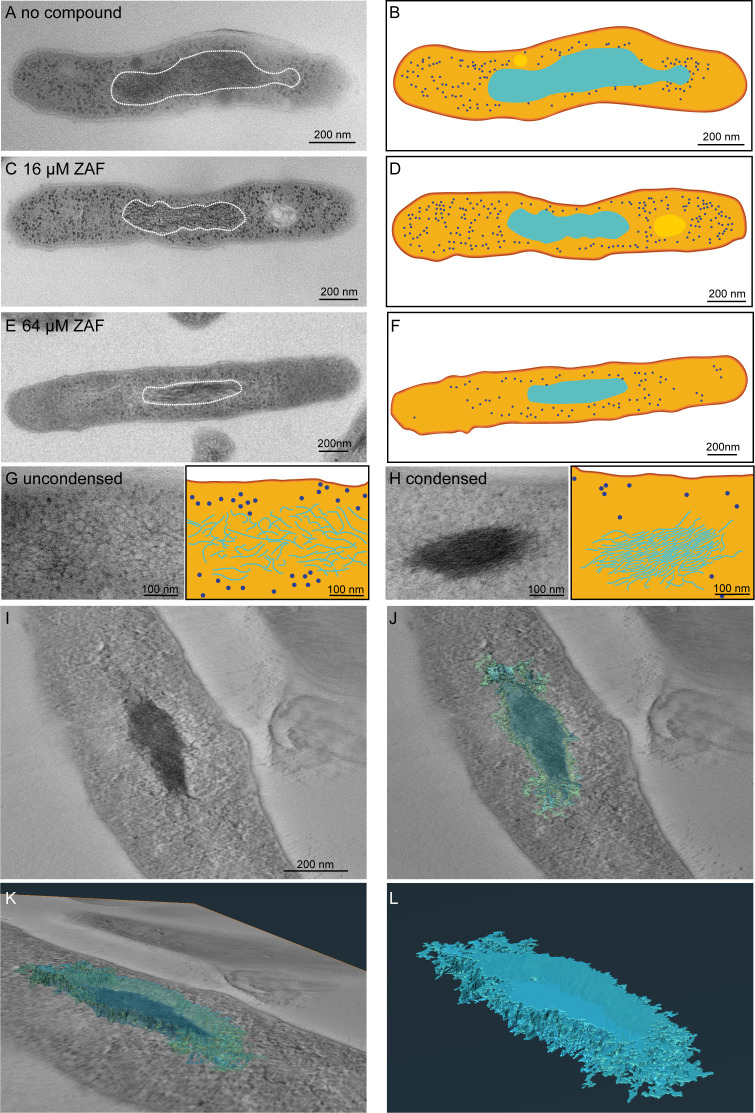
Organization of condensed DNA in *M. abscessus*. Representative TEM images of *M. abscessus* treated with no compound, 16 and 64 µM ZAF. (**A**) One-hour untreated *M. abscessus* have DNA dispersed throughout the majorority of the bacterium. The DNA is present in in the cytosol of the bacterium as electron-dense fiber-like structures. DNA is indicated by a white dotted line. Scale bar indicates 200 nm. (**B**) Schematic representation of A, capsular layer is indicated in dark orange, myco-membrane in light orange, DNA in cyan, and ribosomes in dark blue. (**C**) *M. abscessus* treated with 16 µM ZAF for 1 h. The DNA is present as a small clump of electron-dense fibers close together in the center of the bacterium. DNA is indicated by a white dotted line. (**E**) *M. abscessus* treated with 64 µM ZAF for 1 h. The DNA is present in a small part in the center of the bacterium. DNA is indicated by a white dotted line. scale bar indicates 200 nm. (**F**) Schematic representation of E with the same color code as in B. (**G**) Left panel: high-magnification TEM image of uncondensed DNA in an untreated bacterium. The DNA is present as thin electron-dense fibers with space in between the fibers. The individual DNA fibers have a random orientation. Right panel: DNA fibers are schematically represented with the same color code as B. (**H**) Left panel: high-magnification TEM image of condensed DNA of *M. abscessus* treated with 16 µM ZAF for 1 h. The DNA fibers are densely clumped together, the individual fibers are aligned, and some DNA fibers stick out of the clump. Right panel: organization of the DNA is schematically represented with the same color code as B. (**I–L**) Tomography reconstruction of *M. abscessus* treated with 64 µM ZAF for 1 h. (**I**) Top view of a slice of the tomogram with (**J**) a 3D reconstruction of the DNA on top. DNA is indicated in cyan. (**K**) Side view of 3D-reconstructed DNA from the tomogram displayed in I. (**L**) 3D reconstruction of DNA only, from condensed DNA displayed in I–K.

After showing that ZAF induced DNA condensation as an initial response and altered the localization of DNA into a more dispersed pattern after 1–4 days, we wanted to visualize the effect on the morphology in more detail. *M. abscessus* cultures before and after ZAF treatment were embedded for TEM, which allows analysis with much higher resolution compared to widefield fluorescence. *M. abscessus* treated with 16 and 64 µM and untreated control were imaged using TEM at time points 1 h, 1 day, and 4 days of ZAF treatment. At 1 h, in all three conditions, most of the bacteria were intact and had a homogeneous cytosol with electron-dense DNA and ribosomes (Fig. 4A and B; Fig. S6). The multilayer cell wall was clearly visible in all three conditions with little to no irregularities (Fig. 4A). However, a clear difference was observed in the organization of the DNA. In most of the untreated *M. abscessus*, the DNA was present as electron-dense (black) fibers extending over the entire length of the bacterium (Fig. 4A, indicated by white dotted line and in cyan in 4B). When treated with 16 or 64 µM ZAF, a large proportion of the bacteria had electron-dense (black) fibers clumped together in the middle of the cytosol (Fig. 4C through F; Fig. S6). This is corroborated by our fluorescence microscopy data (Fig. 3). To analyze the organization of condensed DNA in more detail, higher-magnification images are made (Fig. 4G and H). Uncondensed DNA is arranged in electron-dense, thin fibers that are randomly orientated with cytosol in between the fibers (Fig. 4G). Condensed DNA is organized in a clump of electron-dense DNA fibers close to each other with no cytosol in between the fibers. At the outer edge of the DNA clump, some DNA fibers are sticking out (Fig. 4H). To get a more detailed insight in the organization of condensed DNA, we used tomography. With this technique, we were able to study the organization of condensed DNA using a 3D reconstruction of a series of tilted images from a 100-nm section of *M. abscessus* and 3D reconstructing software (Fig. 4I through L; Video S1). We observed that condensed DNA consists of multiple DNA strands that form a single clump of DNA (Fig. 4I and J). In a side view, multiple DNA strands protrude from the DNA clump at different levels of the condensed DNA (Fig. 4K and L), which indicates that condensed DNA is not tightly packed at the exterior of the DNA clump but is tightly packed at the center.

### ZAF affects *M. abscessus* morphology

**Fig 5:**
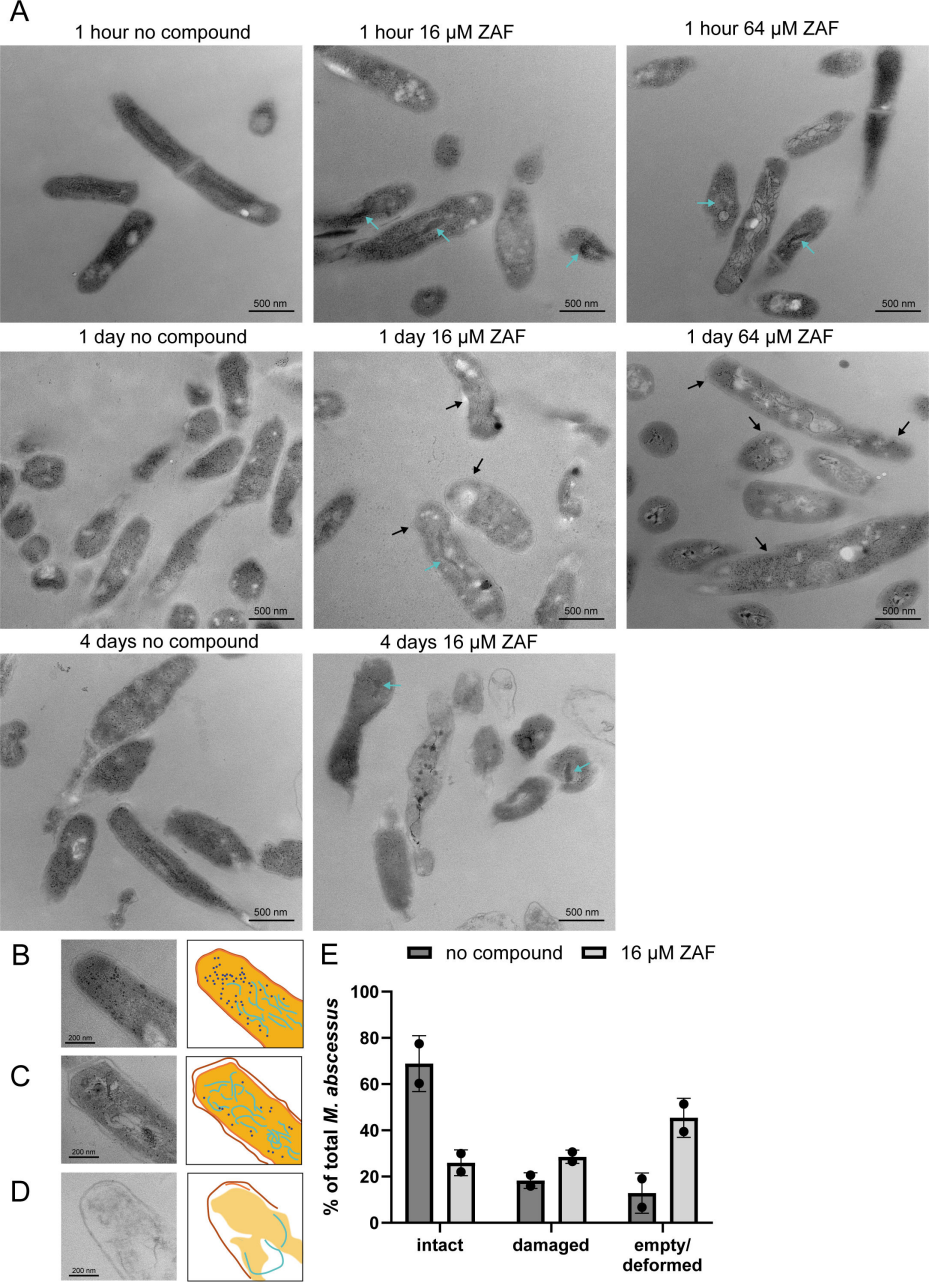
ZAF induces structural abnormalities in *M. abscessus*. (**A**) Representative TEM images of resin embedded *M. abscessus* at 1 h, day 1, and day 4, treated with 16 and 64 µM ZAF. Black arrows indicate membrane damage at 1 day of ZAF treatment, and cyan arrows indicate condensed DNA. All scale bars represent 500 nm. Representative TEM image and schematic representation of (**B**) intact *M. abscessus*, (**C**) damaged *M. abscessus*, and (**D**) empty/deformed *M. abscessus*. Scale bars represent 200 nm. Capsular layer is indicated in dark orange, myco-membrane in light orange, DNA in cyan, and ribosomes in dark blue. (**E**) Quantification of *M. abscessus* morphology at day 4. Untreated *M. abscessus* are indicated in dark grey and 16 µM ZAF in light grey. Bars represent the mean and standard deviation of two experiments.

After 1 day of ZAF treatment, the morphology of *M. abscessus* continued to alter; many of the bacteria were still intact when treated with 16 or 64 µM ZAF (Fig. 5; Fig. S6), but in a small proportion of the bacteria, the outer myco-membrane started to dissociate (indicated by black arrows in Fig. 5A and C). When treated with 64 µM ZAF, the capsular layer and myco-membrane started to become irregular in a larger number of bacteria (indicated by black arrows at Fig. 5A and C; Fig. S6). In addition, “empty” and deformed bacteria were observed at 4 days of 16 µM ZAF treatment (Fig. 5A). Here, the morphology of ZAF-treated *M. abscessus* changed dramatically compared to day 1 and compared to untreated bacteria. The majority of bacteria were either damaged, empty, or deformed, whereas untreated bacteria were mostly intact. The presence of intact, damaged, and empty/deformed bacteria was scored for both 16 µM ZAF-treated and ZAF-untreated *M. abscessus* at Day 4. On average, 69% of the untreated *M. abscessus* were intact: these bacteria had a homogeneous cytosol and a multilayer cell wall with no irregularities (Fig. 5A, B and E). When treated with 16 µM ZAF, 26% of the bacteria had an intact morphology, while 45% of the bacteria were either deformed or empty, indicating that the bacteria were dead or dying (Fig. 5A and B). By comparison, only 13% of untreated *M. abscessus* were deformed or empty. In summary, our TEM analysis showed that ZAF treatment heavily affected the morphology of *M. abscessus*, resulting in damaged or even only remnants of the bacteria at 4 days after treatment.

### ZAF affects ribosome size and localization after 1 day of treatment

**Fig 6:**
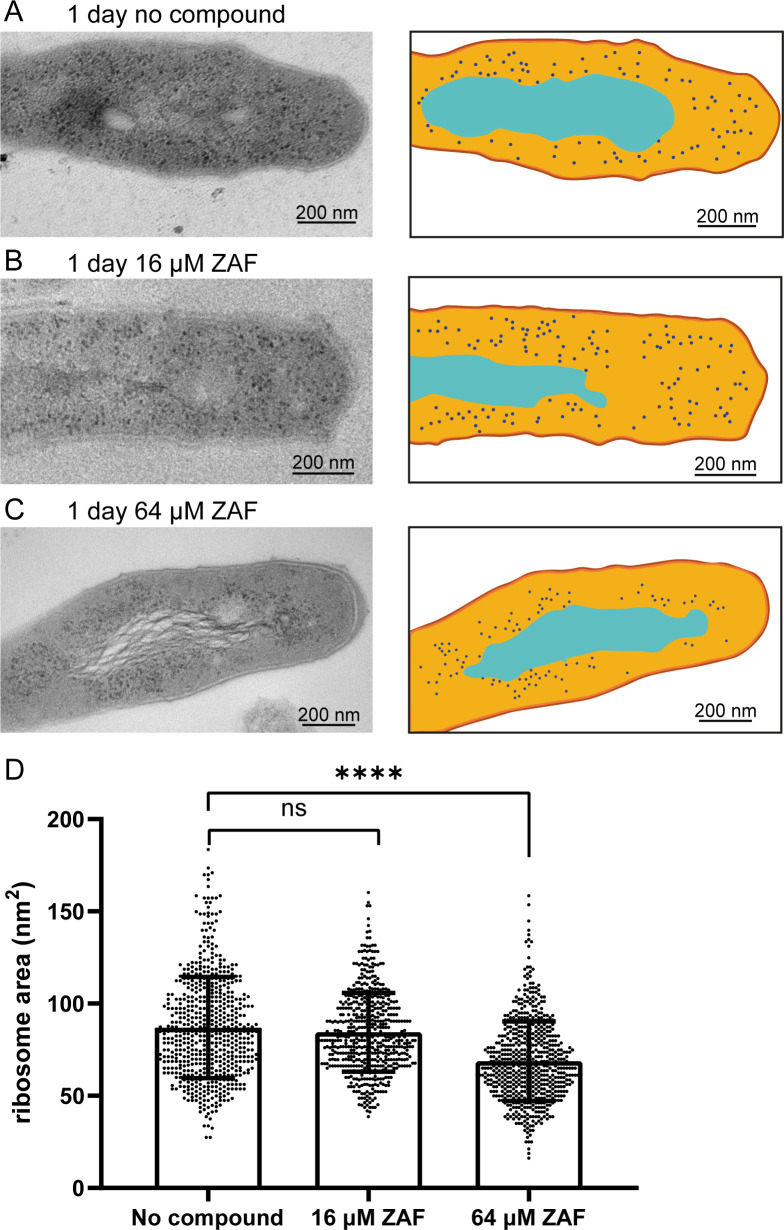
ZAF affects ribosome size after 1 day. Representative TEM image and schematic representation of *M. abscessus* (**A**) 1 day untreated, (**B**) 1-day 16 µM ZAF and (**C**) 1-day 64 µM ZAF. Capsular layer is indicated in dark orange, myco-membrane in orange, cytosol light orange, DNA in cyan, and ribosomes in dark blue. Scale bars represent 200 nm. (**D**) Quantification of the area of the ribosomes from two independent experiments. Per condition, *n* = 174–435 ribosomes were analyzed. A Mann–Whitney test was performed on the data, ****P < 0.0001 and ns = not significant. The graph represents all data points of the two experiments and mean with standard deviation.

We demonstrated that ZAF rapidly affects the structure of DNA in *M. abscessus* and ultimately leads to morphological deformation and cell death. We then wondered if there were additional subcellular defects during the early stages of ZAF treatment, other than DNA relocalization. In *M. abscessus*, it has been shown that the ability to split ribosomes through hflX is correlated with antibiotic resistance (34). From the TEM images, it is clear that in untreated *M. abscessus*, ribosomes are dispersed in the cytosol except for the area of the DNA (Fig. 6A). This was also observed in untreated cultures after 1 h and 4 days of ZAF treatment (data not shown). The dispersed ribosomal localization was also present in *M. abscessus* treated with 16 µM ZAF (Fig. 6B) for 1 day. However, when *M. abscessus* was treated with 64 µM ZAF, ribosomes were clustered closely around the DNA of the mycobacteria (Fig. 6C), leaving a region lacking the electron-dense ribosomes. More detailed analysis showed that the ribosomes were smaller in *M. abscessus* treated with 64 µM ZAF compared to *M. abscessus* treated with 16 µM ZAF and untreated mycobacteria. We measured the area of the ribosomes and determined a significant reduction in ribosomal area (Fig. 6D; Fig. S7). Furthermore, we measured the average ribosome diameter (calculated from the ribosomal area) and found a reduction in diameter from 10 nm in untreated *M. abscessus* to 9 nm in *M. abscessus* treated with 64 µM ZAF for 1 day (Fig. S7). This is in accordance with the previously described ribosomal diameter of *M. tuberculosis*: 10–20 nm (35). Thus, treatment with a high concentration of ZAF affects the localization and size of ribosomes.

## DISCUSSION

In this study, the effect of ZAF on *M. abscessus* growth started to develop after 24–48 h. Importantly, ZAF had an effect on *M. abscessus* cultured in MH2 medium, with an MIC value of 16 µM after 4 days (Fig. 1A). The CFU showed a bactericidal effect at 64 µM after 4 days. This was in line with previous research showing that ZAF treatment reduces *M. smegmatis* growth after 24 h and resulted in complete clearance after 7 days (30). However, the concentration of ZAF necessary for complete clearance of *M. smegmatis* was 10 µM (30)—this is lower than the effective concentration in our study. This suggests that ZAF is more effective in killing *M. smegmatis* than *M. abscessus*. In the study of Pinault et al. (30), *M. smegmatis* was cultured in Luria-Bertani (LB) medium, which was not used in the current study. It is, however, clear that the efficacy of ZAF in killing *M. abscessus* was dependent on the culture medium and ZAF was more efficient in reducing growth of *M. abscessus* in MH2 culture medium than in 7H9 medium. Previous research has shown that culture medium affects *M. abscessus* colony morphology and virulence (36). It could be that in our study, the culture medium affected the characteristics of *M. abscessus* and thereby ZAF functioning, since the rough colony variant has higher Lsr2 expression than the smooth colony variant (27). In addition, it is possible that ZAF bound to a component that is present in 7H9 and not in MH2 culture medium, resulting in reduced efficacy of ZAF. Our findings are in contrast with those of Lavollay et al. (37), who found a significant decrease in the MIC of cefoxitin and IPM on clinical isolates when cultured in MH2 medium compared to 7H9. Adding albumin to the culture medium rendered ZAF to be ineffective, and the bactericidal effect on *M. abscessus* was completely abrogated. Indeed, others have shown that ZAF can bind to plasma proteins like albumin (31). As albumin is abundant in blood and tissues, this is clearly a practical problem when ZAF is applied as an antibiotic strategy. Therefore, we are currently working on chemically altering the structure of ZAF to reduce binding without affecting the bactericidal effects of ZAF. In addition to albumin, adding DC to culture medium resulted in the reduced efficacy of ZAF. Interestingly, these compounds also induced smooth colony growth of *M. abscessus*, in which less Lsr2 is expressed compared to rough colonies (27, 36).

We showed for the first time that treatment with ZAF initially causes condensation of *M. abscessus* DNA at 1 h. Even though *M. abscessus* is an NTM, its response to antibiotic stress was the same as we and others have described for *M. tuberculosis* and *M. smegmatis* (14). As for *M. tuberculosis* and *M. smegmatis*, DNA condensation was primarily detected in live bacteria and less evident after bacteria were killed. In this study, we found that the morphology of *M. abscessus* was already altered after 1 h and even further at 1 day of 64 µM ZAF treatment and after 4 days of 16 µM ZAF treatment. The effect of ZAF treatment can be broader as others have shown that Lsr2 binds around 21% of the *M. tuberculosis* genome, affecting genes involved in cell wall synthesis, virulence factors, and genes involved in the stress response (19). In *M. smegmatis*, deletion of Lrs2 results in different colony morphology, rendering the bacteria unable to form biofilms, and also results in shorter more irregularly shaped bacteria (15, 16, 23). Taken together, it is possible that the effect of ZAF might extend further than on DNA condensation alone. We have demonstrated that recovery from condensation of the DNA can be exploited as antibiotic strategy (14) and renders *M. tuberculosis* more sensitive to antibiotics. Here, we demonstrated that *M. abscessus* was vulnerable to a single dose of ZAF. Our detailed ultrastructural analysis highlights the effects of ZAF on DNA and the size and localization of ribosomes but also on the capsular layer and the entire cytosol, eventually leaving the bacteria completely damaged and as an empty ghost.

It is exciting to realize that ZAF treatment alone had a bactericidal effect, specifically as ZAF is an FDA-approved compound. Pinault et al. already proposed to use the compound on mycobacterial infections and recently Ngidi et al. (38) proposed to use Accolate (brand name of ZAF) as an *M. tuberculosis* antibiotic, as it binds *in silico* with high energy to the drug target: fatty acid degradation protein D32. In addition, ZAF was shown to have a maximum affinity toward enzymes essential for *Leishmania* parasite survival (39). Furthermore, ZAF inhibits biofilm formation and viability of *P. gingivalis* and *Streptococcus mutans*, at a concentration of 50 µM (40). Newly synthesized analogs of ZAF even have improved activity in *P. gingivalis* (32); however, five of these analogs tested did not improve mycobacterial killing. ZAF has also been applied to mycobacterial infections; in a clinical trial where ZAF treatment was administered to patients with *Mycobacterium leprae* reactions, ZAF was effective in reducing symptoms in 92% of patients (41). Further research is needed to determine the effect of ZAF on *M. abscessus*-infected patients, and a good start might be the study of infected macrophages. Deletion of Lsr2 resulted in reduced survival of *M. smegmatis* and *M. abscessus* in murine macrophages (20, 27). Moreover, deletion of Lsr2 in *M. abscessus* resulted in decreased survival in zebrafish embryos and Balb/c mice (27). However, it is possible that ZAF itself affects macrophages, since these cells do express the cysteinyl leukotriene receptor, which is the target of ZAF in asthma treatment. Leukotrienes enhance bacterial killing of *K. pneumoniae* by alveolar macrophages (42).

To our knowledge, ZAF has not been clinically applied to any bacterial infection. This could be due to the relatively high doses that have to be administered to have a complete bactericidal effect. The MIC of ZAF in *in vitro* cultures is 16 µM (i.e., 9.21 µg/mL). For asthmatic applications, ZAF is administered orally at a daily dose of 20 mg, and the maximum recommended daily oral dose is 10 mg/kg/day. Importantly, the terminal half-life of ZAF is 10 h. Therefore, whether this dose is applicable to large groups of patients needs to be determined. It is important to point out that ZAF appears to be only effective on replicating *M. abscessus* and not in persisting/starvation conditions. Our data also show no synergy with other antibiotics. This, together with the limitations in culture conditions, makes ZAF not ready for broad clinical applications. Still, when applied to the extensively drug-resistant *M. abscessus*, the drug may be a useful addition to the current insufficient drug regimen. In this study, we have shown that under specific conditions, the anti-asthma drug ZAF kills *M. abscessus* in a dose-dependent manner. Since ZAF is FDA-approved, it is an interesting avenue of research to pursue. By repurposing this drug, it could be applied to treat extensively drug-resistant *M. abscessus* infections.

## MATERIALS AND METHODS

### Bacterial strains

*M. abscessus* ATCC 19977, *M. intracellulare* ATCC 13950, and *M. avium* ATCC 25921 were purchased from the American Type Tissue Collection (ATCC, Manassas, VA, USA). *S. enterica* ATCC 14028, *K. pneumoniae* ATCC 27736, *A. baumannii* ATCC 19606, and *E. coli* were obtained from Prof. Paul Hergenrother (University of Illinois Urbana-Champagne). *P. aeruginosa* ATCC 27853, and *E. cloacae* ATCC 13047 were from Prof. Dev Arya (Clemson University).

### Determination of MIC values of ZAF and its analogs against various mycobacterial strains

Antibacterial activity was determined using the standard double-dilution method. *M. abscessus* ATCC 19977 was grown in Mueller–Hinton (MH) broth 2 (MH2, Sigma-Aldrich #90922), MH2 with 10% DC, MH2 with 10% ADC, 7H9, 7H9 with 10% DC, or 7H9 with 10% ADC. DC contained 20-g/L dextrose, 40-mg/L catalase, and 8.5-g/L NaCl, while ADC additionally contained 50-g/L bovine serum albumin (BSA, Fraction V). All other mycobacteria were grown in 7H9 broth alone or supplemented with 0.05% Tween 80, 0.4% glycerol, and 10% ADC. The mycobacterial strains were grown on agar plates corresponding to the growth medium (e.g., *M. abscessus* on 7H9 agar supplemented with 0.05% Tween 80 and 0.4% glycerol, and all other mycobacteria on 7H10 supplemented with 0.05% Tween 80, 0.4% glycerol, and 10% OADC (ADC with 0.5-g/L oleic acid)). Cells were transferred from the plates to the corresponding liquid medium until the attenuance at 600 nm was equal to that of a 0.5 McFarland standard. The culture was then diluted 1:100 and added to the compounds dissolved in the medium corresponding to the one used for bacterial species and the experiment. Plates were incubated at 37°C until growth was observed in the wells containing no compound (3 days to 3 weeks, mycobacterial species-dependent). To positively assess growth inhibition, 5 µL of a 2.5-mg/mL solution of the live/dead stain resazurin (also known as alamarBlue) was added to each well prior to overnight incubation at 37°C. Time-course monitoring of 96-well plates was done on a SpectraMax M5 microtiter plate reader at 600 nm, and readings were taken at the indicated time points (e.g., 1, 2, 3, and 4 days).

### Determination of MIC values of non-mycobacteria

Bacterial strains were plated on MH agar. As with the mycobacteria, enough bacteria were scraped from the plate to make a culture of OD_600_ 0.5. Bacteria were diluted 1:1,000 to make a working culture, and that culture was further diluted 1:1 with medium-containing compound in a 96-well format. Bacterial growth was observed after 16 h.

### Combination treatment of *M. abscessus* with ZAF and antibiotics

*M. abscessus* was treated identically to the original MIC determination. For combination treatment, antibiotics were diluted horizontally on individual 96-well plates, and ZAF (0, and 8 μM to 128 µM) was diluted vertically. The antibiotics used include IPM (0 and 0.1 μg/mL to 12.8 μg/mL), KAN (0 and 0.013 µg/mL to1.6 μg/mL), RIF (0 and 0.1 μg/mL to 12.8 μg/mL), cefotaxime (CTX, 0 and 0.051 μg/mL to 6.4 μg/mL), and tigecycline (TG, 0 and 0.003 μg/mL to 0.4 μg/mL). No combination resulted in a change of the ZAF MIC value.

### Preparation of *M. abscessus* for microscopic analysis

*M. abscessus* was scraped from an MH agar plate and suspended in MH2 (or 7H9) medium. The culture was diluted to an OD_600_ (as measured by densitometer) of 0.4–0.5. ZAF at its desired concentrations was added to the cultures. The cultures (5–10 mL) were grown at 37°C for the times indicated in Fig. S1. OD_600_ measurements were taken every 24 h using the same densitometer as above.

### CFU determination

Bacteria were diluted as for MIC determination into 5-mL tubes of MH2. To this culture was added dimethyl sulfoxide (DMSO) (untreated), 16 or 64 µM ZAF, and cultures were incubated at 37°C. OD_600nm_ and a 100 µL sample of the culture were taken every 24 h at times of 0, 24, 48, 72, 96, and 168 h. Samples were subjected to a 10-fold dilution in MH2, and 100 µL of the appropriate dilutions were plated on 7H10 agar. After 4 days of growth, colonies were counted, and the CFU/mL was calculated (Fig. S4A).

### Treatment of non-replicating *M. abscessus* for CFU

*M. abscessus* was scraped from an MH agar plate and suspended in MH2 medium. The culture was diluted to an OD_600_ of 0.5. To mimic non-replicating conditions (33), the bacteria were pelleted by centrifugation (3,500 rpm, 15 min) and washed with PBS (5 mL) twice before a final resuspension in PBS (5 mL). The appropriate amount of ZAF was then added. The cultures were grown and sampled as in the CFU determination section. CFU results can be seen in Fig. 2B.

### Chemical fixation of treated bacteria

Bacteria from the 5- to 10-mL cultures described above were pelleted at 3,500 rpm for 20 min at 4°C. The medium, with exception of 0.5 mL, was removed. Fixative (0.5 mL of 11.5:12.5:1/buffer A:8% paraformaldehyde:25% glutaraldehyde) was added and incubated at room temperature for 4 h. Buffer A consisted of 0.24-M PIPES, 0.1-M HEPES, 8-mM MgCl_2_, and 40-mM EGTA, at a pH of 6.9 (adjusted at room temperature). The bacteria were pelleted again (3,500 rpm, 20 min, room temperature) and gently resuspended in 0.5-mL storage buffer (5:1.2:13.8/buffer A:8% paraformaldehyde:sterile ddH_2_O). A small sample (10 µL) was plated on MH agar to confirm that no living bacteria remained. Samples were stored at 4°C until analyzed.

### Imaging and quantification of DNA condensation in *M. abscessus*

Fixed *M. abscessus* cultures were stained with Nile red (1:50, 1 mg/mL in EtOH, Sigma 72485) to stain lipids and Hoechst (1:250, Invitrogen 33342) for 10 min. The stained mycobacteria were then centrifuged at 5,000 rpm for 5 min and washed twice with ddH_2_O. After centrifugation at 5,000 rpm for 5 min, the supernatant was removed, and 50 µL of pellet was left in the Eppendorf tube. Stained *M. abscessus* were incubated on poly-l-lysine (0.1 mg/mL in ddH_2_O)-coated glass slides for 20 min in the dark at room temperature. Thereafter, the excess cell suspension was slowly removed, and *M. abscessus* was mounted with Vectashield and a coverslip. The stained mycobacteria were imaged using a Leica DM6 widefield fluorescence microscope using excitation 350/50 and emission 460/50 to detect Hoechst and excitation 545/25 and emission 605/70 to detect Nile red. The images were quantified using ImageJ Fiji. Deconvolution was performed using Huygens deconvolution software (Huygens Deconvolution | Scientific Volume Imaging (svi.nl)). To quantify the percentage of condensed DNA, the morphology of the DNA was assessed. The DNA was considered condensed when the DNA was present as a round, small clump in the center of the mycobacterium. Nile red was used to indicate the length of the mycobacterium, as it stains the lipids present in the cell wall of the mycobacterium. Per condition, 40–479 individual mycobacteria were scored, and the percentage of mycobacteria with condensed DNA was calculated per condition for two to three experiments.

### Transmission electron microscopy

Fixed *M. abscessus* cultures in storage buffer were washed with H_2_O by centrifugation at 5,000 rpm for 5 min and incubated for 1 h in osmium tetraoxide (1% in ddH_2_O). Thereafter, the bacteria were washed with H_2_O and dehydrated with increasing concentrations of EtOH. *M. abscessus* were incubated twice with 70% EtOH for 15 min, with 80% EtOH, with 90% EtOH, and twice with 100% EtOH. After each step, the bacteria were centrifuged at 5,000 rpm for 5 min, and the supernatant was removed. In addition, *M. abscessus* was infiltrated for 2 h with 1:1 epon (electron microscopy sciences) and propylene oxide, followed by 2 h with epon. Lastly, the epon was refreshed, and the samples were polymerized at 60°C for at least 2 days. The samples were cut using a diamond knife (Diatome) on a Leica Ultracut U7 microtome, and sections were contrasted with uranyl acetate (3.5% in ddH_2_O) and lead citrate (Electron Microscopy Sciences, cat #22410). The samples were imaged using a Tecnai 120-kV microscope with a Veleta and Xarosa camera. Images were analyzed using ImageJ Fiji.

### Tomography

Sections of 100-nm-thick were cut from epon-embedded *M. abscessus* using a diamond knife (Diatome) on a Leica Ultracut U7 microtome and contrasted with uranyl acetate (3.5% in ddH_2_O) and lead citrate (Electron Microscopy Sciences, cat #22410). The grids were then incubated with protein A 10-nm gold (Utrecht University), which acts as a fiducial marker, for 1 min, and the excess was blotted away. Tomography was performed with a Talos L120C electron microscope at 120 kV with a Ceta 16 M camera in a ±60–65° tilt series with 1° increments. Alignment was performed using Inspect3D software (Thermo Fisher Scientific). In short, tracking was based on 16 patches or gold beads, and the gold beads were cloaked afterward. The tomogram was reconstructed by Simultaneous Iterative Reconstruction Technique with 20–60 iterations. Surface modeling and reconstruction were performed using Amira software (Thermo Fisher Scientific).

### Statistics

Data were analyzed using GraphPad Prism (version 9.3.1). The quantification of DNA condensation was tested for normal distribution using the Shapiro–Wilk test. The data were considered normally distributed. An unpaired *t*-test was performed on the DNA condensation and absorption data. The ribosome data were not normally distributed; therefore, a Mann–Whitney test on the pooled data of the two experiments was performed. *P < 0.05, **P < 0.01, ***P < 0.001, ****P < 0.0001.

## SUPPLEMENTAL MATERIAL

Supplemental material aac.00029-24-s0001.docx Table S1, Figures S1 to S7, and legend for Video S1.

Video S1 aac.00029-24-s0002.mp4 TEM tomogram stack of 100-nm-thick section of *M. abscessus* treated with 64 µM ZAF for 1 h.

ASM does not own the copyrights to Supplemental Material that may be linked to, or accessed through, an article. The authors have granted ASM a non-exclusive, world-wide license to publish the Supplemental Material files. Please contact the corresponding author directly for reuse.
