# Supplementary material for: Zafirlukast induces DNA condensation and has bactericidal effect on replicating Mycobacterium abscessus
Source: Antimicrob Agents Chemother. 2024 Jul 11;68(8):e00029-24. doi: 10.1128/aac.00029-24 (PMC11304721; doi:10.1128/aac.00029-24)
Supplement: Supplemental material — Table S1, Figures S1 to S7, and legend for Video S1. [file aac.00029-24-s0001.docx]

**Zafirlukast induces DNA condensation and has bactericidal effect on replicating *Mycobacterium abscessus***

Sanne van der Niet^1^, Keith D. Green^2^, Irene M. Schimmel^1^, Jordy de Bakker^1^, Bastiaan Lodder^1@^, Eric A. Reits^1^, Sylvie Garneau-Tsodikova^2^ and Nicole N. van der Wel^1*^

^1^Electron Microscopy Centre Amsterdam, Amsterdam University Medical Centre, the Netherlands

^2^College of Pharmacy, University of Kentucky, Kentucky, United States of America

*Corresponding author, email address: [n.n.vanderwel@amsterdamumc.nl](mailto:n.n.vanderwel@amsterdamumc.nl)

Present address:

# National Institute for Public Health and the Environment

@ Netherlands Institute for Neuroscience

**SUPPLEMENTAL TABLE**

| **Supplemental Table 1.** MIC (μM) of ZAF with non-mycobacteria. | | | |
| --- | --- | --- | --- |
| Bacterial strain | ZAF | AMK | KAN |
| *S. enterica* ATCC 14028 | >128 | 1 | 4 |
| *K. pneumoniae* ATCC 27736 | >128 | 0.5 | 2 |
| *A. baumannii* ATCC 19606 | >128 | 2 | 8 |
| *E. coli* MC1061 | >128 | ≤0.25 | 0.5 |
| *P. aeruginosa* ATCC 27853 | >128 | 0.5 | >128 |
| *E. cloacae* ATCC 13047 | >128 | ≤0.25 | 2 |

**SUPPLEMENTAL FIGURES**


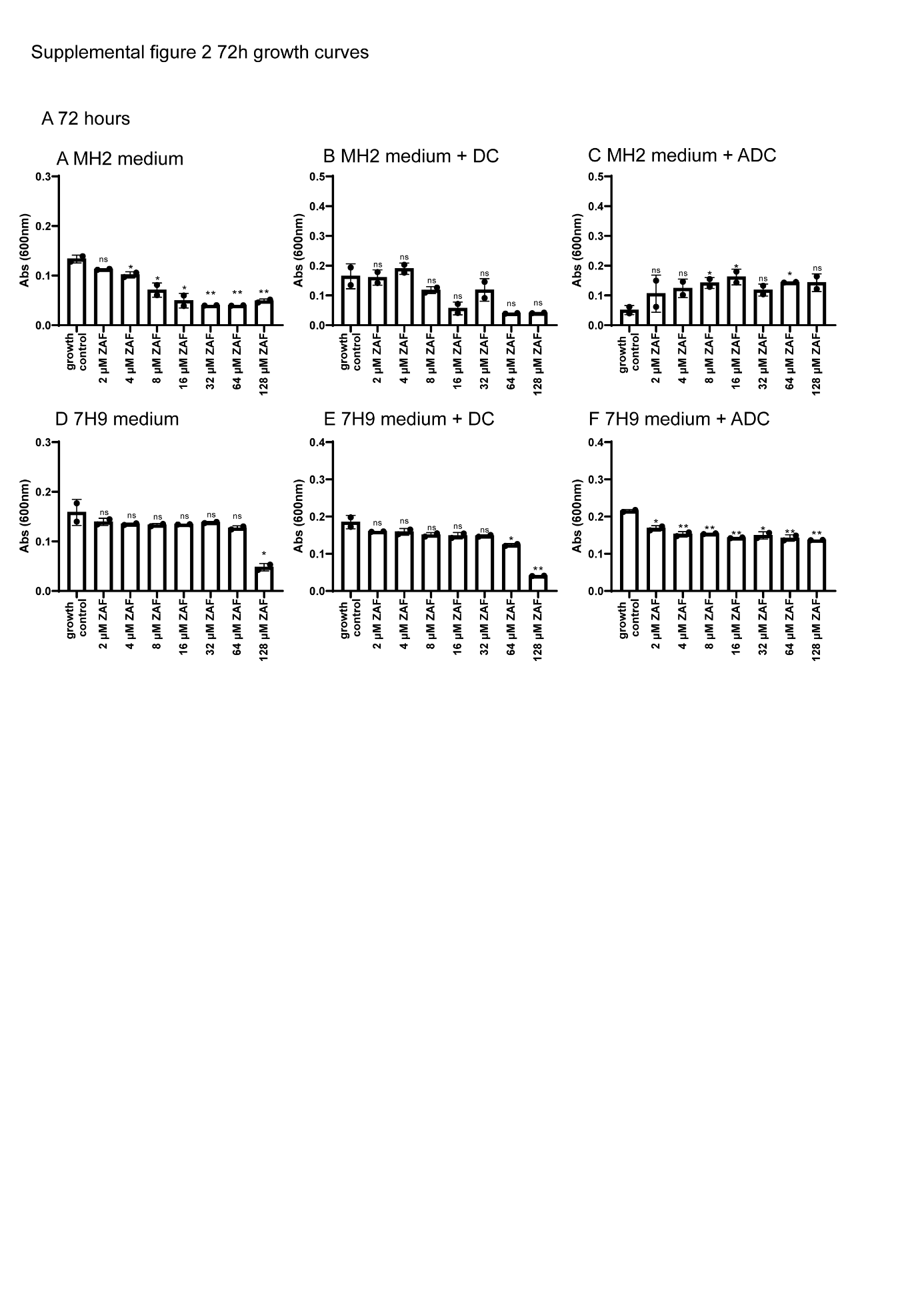


**Figure S1: Effect of ZAF on *M. abscessus growth* in different culture media after 72 h.** Absorption values of *M. abscessus* at 72 h of treatment with 2, 4, 16, 32, 64, and 128 µM ZAF. *M. abscessus* was cultured in **A.** MH2 medium, **B.** MH2 medium supplemented with DC, **C.** MH2 medium supplemented with ADC, **D.** 7H9 medium, **E.** 7H9 medium supplemented with DC, and **F.** 7H9 medium supplemented with ADC. Bars represent the mean of two individual experiments and the error bars represent the standard deviation. * p <0.05, ** p <0.01, and ns indicates not significant.


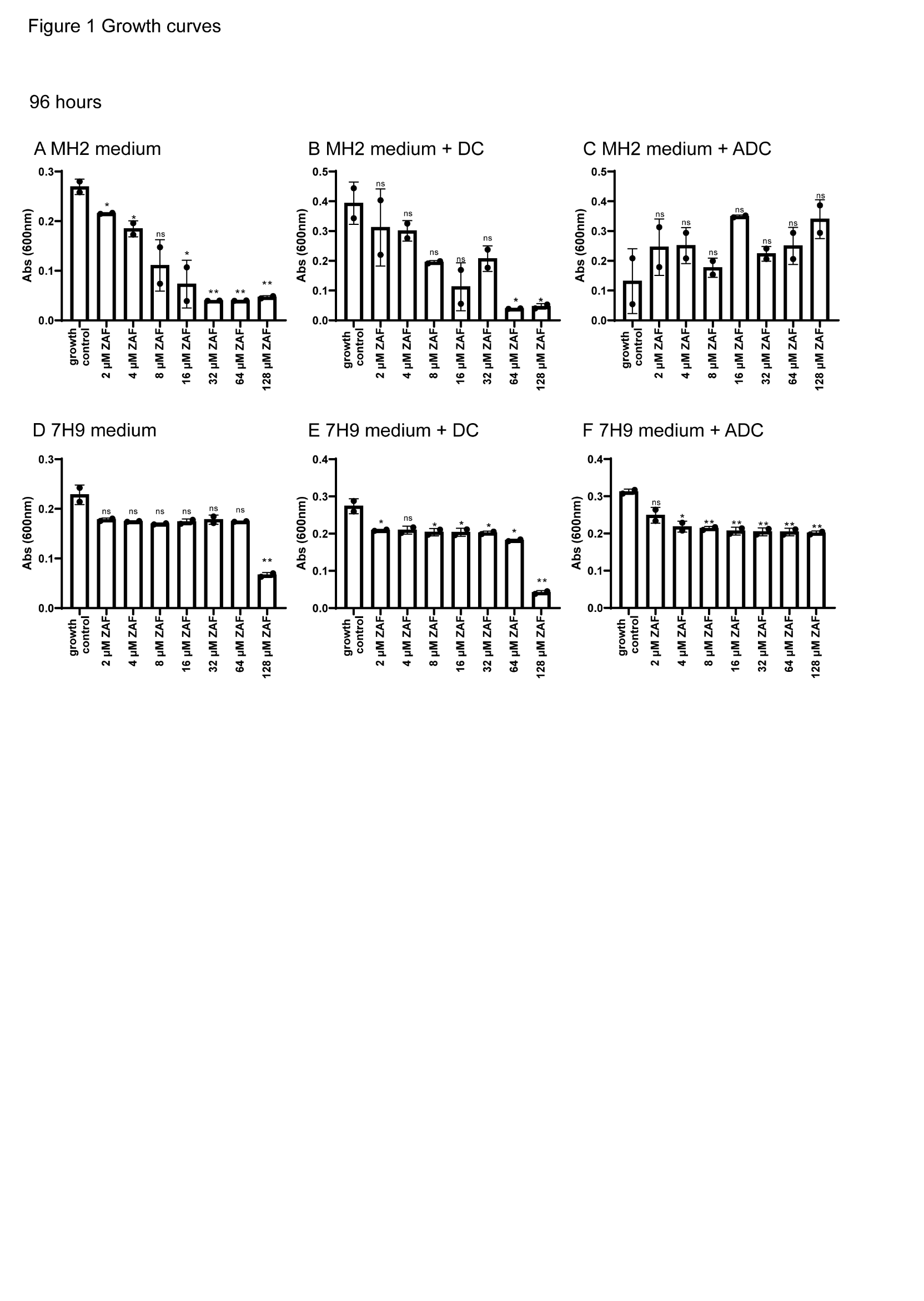


**Fig S2:** **Effect of ZAF on *M. abscessus* growth in different culture media.** Absorption values of *M. abscessus* at 96 h of treatment with 2, 4, 16, 32, 64, and 128 µM ZAF. *M. abscessus* was cultured in **A.** MH2 medium, **B.** MH2 supplemented with DC, **C.** MH2 medium supplemented with ADC, **D.** 7H9 culture medium, **E.** 7H9 supplemented with DC, and **F.** 7H9 supplemented with ADC. Bars represent the mean of two individual experiments with the standard deviation. * p <0.05, ** p <0.01, and ns indicates not significant.


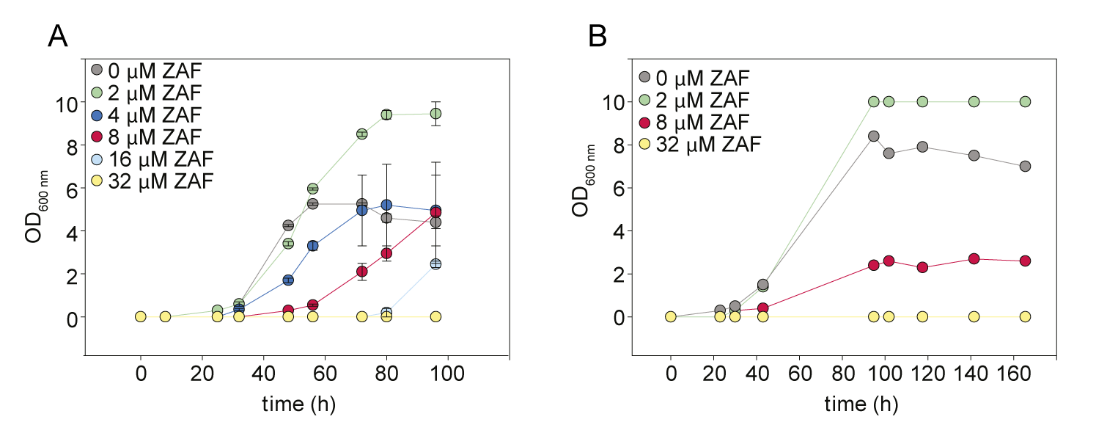


**Figure S3: Growth curves of *M. abscessus* during 2 additional experiments in MH2 medium. A.** *M. abscessus* was treated with 0 (grey), 2 (green), 4 (dark blue), 8 (red), 16 (light blue), and 32 (yellow) µM ZAF and the absorption was measured every 24 h for a period of 168 h. Error bars show the variation in duplicates of the cultures treated with corresponding concentrations of ZAF. **B.** *M. abscessus* was treated with 0 (gray), 2 (green), 8 (red), and 32 (yellow) µM ZAF and the absorption was measured every 24 h for a period of 168 h. These experiments were performed in 5 mL volumes while the experiments presented in Figure 1 (in the main text) were performed in 96-well plates and a volume of 200 μL.

**Figure S4: Minimum inhibitory concentrations of ZAF in combination with antibiotics.** *M. abscessus* was treated with ZAF in combination with cefotaxime (CTX), imipenem (IPM), kanamycin (KAN), rifampicin (RIF), and tigecycline (TG), and the minimum inhibitory concentration (MIC) values were determined.


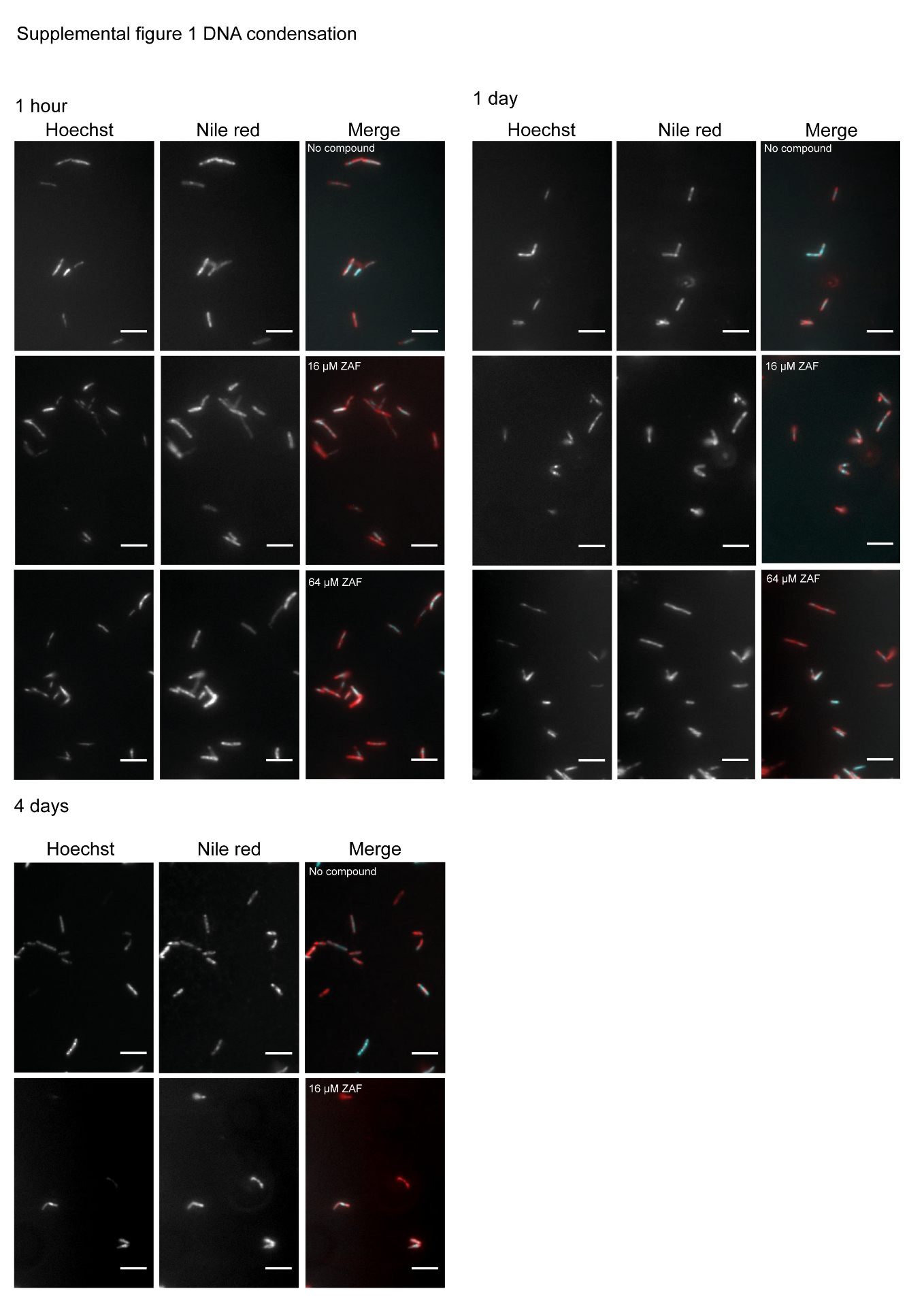


**Figure S5:** **ZAF induces rapid DNA condensation.** Representative widefield fluorescent images of *M. abscessus* treated with no compound, 16 µM ZAF, or 64 µM ZAF for 1 h, 1 day, and 4 days. DNA was stained with Hoechst (cyan) and lipids with Nile red (red). Hoechst and Nile red channels are shown separately in greyscale and the merged, colored, images are displayed. White arrows indicate condensed DNA. Scale bars represent 5 µm.


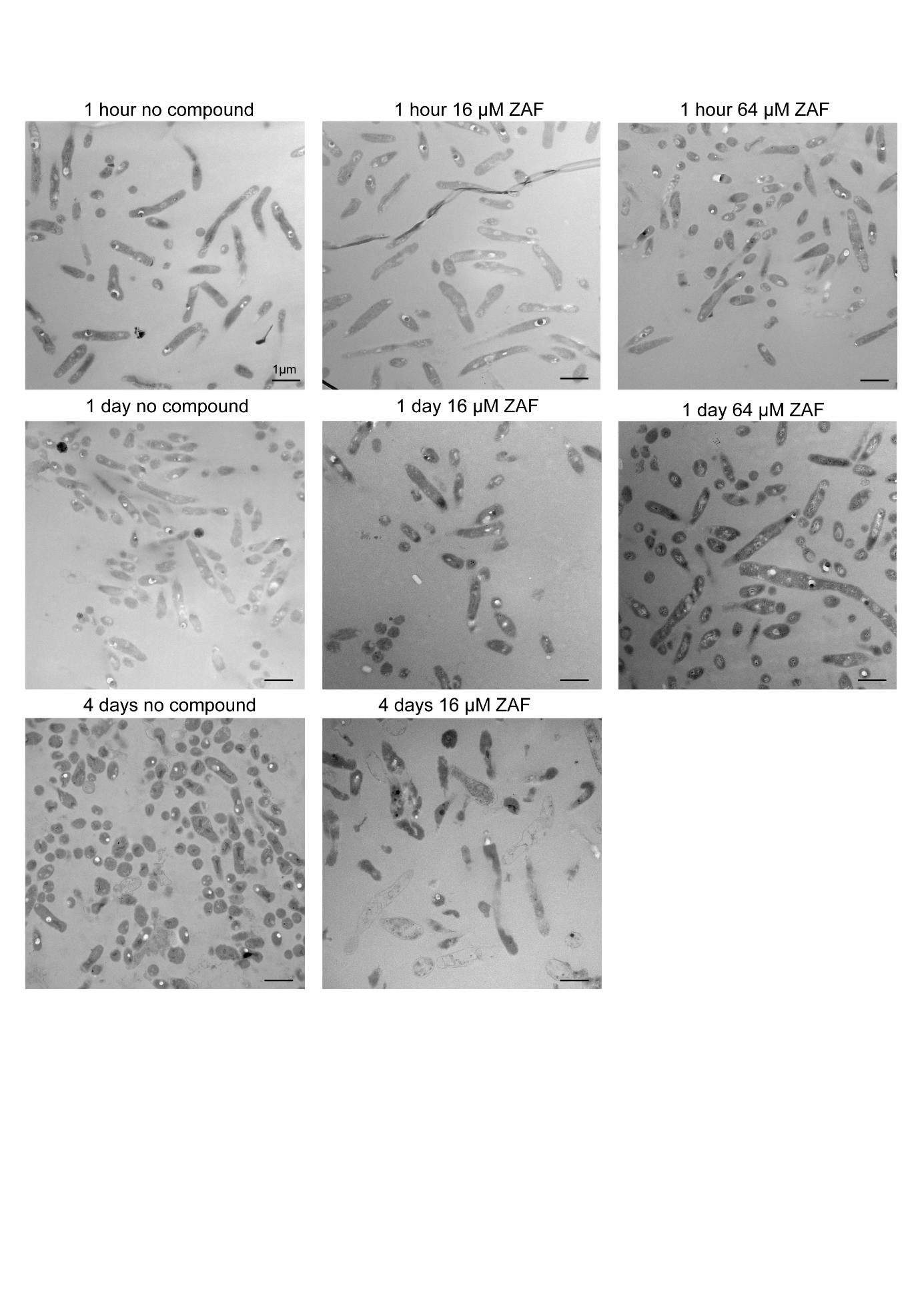


**Figure S6: ZAF affects *M. abscessus* morphology.** Representative low magnification TEM images of resin embedded *M. abscessus* at 1 h, day 1, and day 4, treated with 16 µM or 64 µM ZAF, and untreated control. Scale bars represent 1 µm.


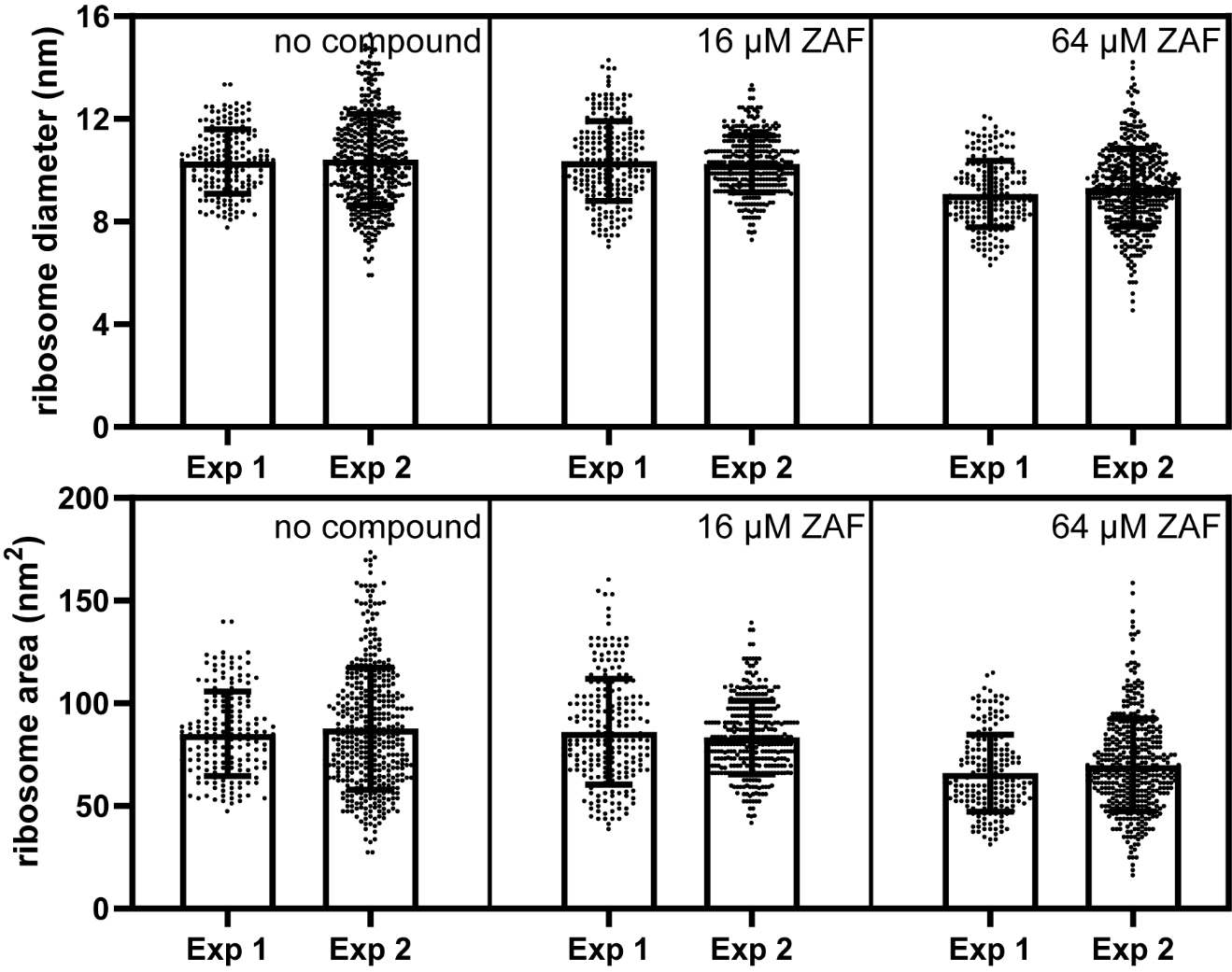


**Figure S7:** **Ribosome size is affected by ZAF after 1 day.** The size of ribosomes were quantified in TEM images of *M. abscessus* not treated or treated for 1 day with 16 or 64 µM ZAF. The size and diameter of the ribosomes in 2 independent experiments were measured. Per condition, n = 174-435 ribosomes were analyzed.

**Supplemental Video 1**: TEM tomogram stack of 100 nm thick section of *M. abscessus* treated with 64 µM ZAF for 1 hour.
